# Inter-organizational conflict and construction project performance: Influencing mechanism based on trust networks

**DOI:** 10.1371/journal.pone.0331014

**Published:** 2025-08-29

**Authors:** Xiang Wang, Yilin Yin

**Affiliations:** 1 School of Management Engineering, Qingdao University of Technology, Qingdao, Shandong, P. R. China; 2 College of Management, Tianjin University of Technology, Tianjin, P. R. China; Islamic Azad University Urmia Branch, IRAN, ISLAMIC REPUBLIC OF

## Abstract

Research on inter-organizational conflicts among the participants of construction projects typically concentrate on the binary perspective of the project owner and contractor, disregarding the correlation of the complex relationship networks on conflict incidents. This research will propose a systematic set of strategies for reducing inter-organizational conflicts and enhancing project performance from the perspective of trust networks governance. Specifically, the research aims to uncover the interactive mechanisms among the trust network of all participants in construction projects, inter-organizational conflicts, and project performance, thereby filling the existing research gaps. The PLS-SEM approach was employed to analyze 207 valid questionnaires from the participants of construction projects. Data analysis revealed that both the density and stability of the trust networks can restrain various forms of inter-organizational conflicts, and inter-organizational conflicts can impede the attainment of project performance. The density of trust networks can enhance project performance, yet the extent to which the stability of network structure is related to this aspect remains unproven. These findings are conducive to optimizing the performance level of engineering projects and reducing the probability of inter-organizational conflicts.

## Introduction

Inter-organizational conflicts are an inescapable practical issue throughout the entire implementation process of construction projects. The effective management and control of inter-organizational conflicts constitutes a crucial task in modern project governance during practical operations. In the course of enhancing the cooperation efficiency among project participants, promoting the improvement of project performance and the realization of project value, previous research have attested to the unique role of inter-organizational conflicts from the binary perspective of the owner and contractor. Nevertheless, when the solution approach of the social network theory gains attention and importance from project managers, it enters a brand-new realm of diversified governance. In this context, traditional mechanisms such as contractual governance and relational governance, being constrained by a binary analytical basis, demonstrate insufficient explanatory power when confronted with numerous project participants or stakeholders. That is to say, although prior reserach have indicated that contract – related or relationship – governance factors such as contract rights, contract complexity, and organizational culture can alter the likelihood of inter – organizational conflicts arising or the degree of their interference in engineering projects, they have overlooked the roles of numerous participants in construction projects. Consequently, how to inhibit the occurrence of conflicts among these numerous project participants or decrease the degree of conflict relevance remains an area yet to be fully explored.

It is gratifying that previous research have indicated that the networked structure of trust relationships among project participants constitutes a distinctive project governance mechanism, namely the trust networks. It is executed in accordance with the project management outline or the engineering management implementation plan, enabling numerous project participants of a project to coalesce based on trust relationships and exert its unique role centering around the enhancement of project performance and the realization of engineering value. Previous studies that have been concerned with project owners and contractors in engineering projects have underscored the positive correlation of trust relationships in facilitating the favorable development of inter – organizational conflicts. However, these studies have not specified whether the trust relationships among other project – participating parties can optimize the climate of inter – organizational conflicts. Thus, this research endeavors to analyze the complex relationships among trust networks, inter-organizational conflicts, and project performance, offering diversified governance paths for the smooth implementation and performance improvement of engineering projects.

### Theoretical background

#### Trust network structure among project participants.

Numerous research on trust have been performed, and the definitions, conclusions, and related inferences have been discussed in depth in the fields of psychology, sociology, management, and computer science [1]. This provides more humanized theory and method support for the harmonious and efficient development of economic society [2]. This research focused on the trust network structure among the participants of construction projects. However, existing research in this area has mainly focused on the dual trust relationship perspective between owner and contractor. In other words, previous research have ignored the complex social networks embedded in the many project participants and stakeholders [3].

Fortunately, previous research in other areas have defined the trust networks as a network of relationships, in which people are willing to put valuable, important, long-term resources, or businesses at risk of other people’s bad behaviour. On this basis, some researchers have analysed the trust networks from the perspective of public governance and informal system. In addition, some research have focused on the relationship of trust networks with higher education, but not on firms or organisations management [4]. Further, some research in the supply chain field demonstrate the relationship of trust networks between firms on their opportunistic behaviour by portraying the network structure as a complex adaptive system. However, the interpretation and analysis of trust networks mainly focus on cryptography, computer science, and other fields and on the optimization of trust algorithms or the decentralization of trust networks.

Therefore, previous reserach have failed to explain the network structure of trust relationships among participants in construction projects and its associated mechanisms with the projects [5]. In fact, many participants in an construction project need to work together to complete the task, but their interests, emotional expectations, working methods and attitudes are completely different, so the stable cooperation between these participants through the trust relationship link is crucial to the success of the construction project. The core reason is that because each participant has different understanding and execution power of rigid economic contract with the owner of the project, trust relationship as a flexible mechanism can fundamentally make up for the details that cannot be taken into account by contract terms [6]. Unfortunately, previous research in this field is relatively insufficient, resulting in the lack of theoretical and methodological support in the aspects of communication, coordination, win-win cooperation and other aspects of the participants in construction projects [7].

### Inter-organizational conflict of project participants

Inter-organizational conflict, a common occurrence during the collaboration process among project participants, pervades all aspects of the entire life cycle of a construction project. For the purpose of facilitating research and analysis, scholars have provided diverse definitions of inter-organizational conflict from varying perspectives [8]. Early research defined inter-organizational conflict as an interaction process of disputes, disharmony, or discord among groups. Alternatively, it is a hostile sentiment, representing a state of incongruence regarding goals and values among two or more participants in a certain relationship. It can also be considered as a phenomenon where two or more participants have completely distinct ideas, beliefs, and interests. Recent relevant research have reached a convergence in the definition of conflict. Inter-organizational conflict is a state form or an atmosphere of disharmony, arising from the differences in goals, cognition, or emotions among two or more participants [9]. In reality, inter-organizational conflict can hardly be eradicated completely. Hence, two or more participants can manage conflict incidents during the collaboration process in order to facilitate the successful attainment of the cooperation goals.

In fact, numerous factors can be associated with inter-organizational conflicts in construction projects. Past research has yielded a great many profound findings in this area. Nonetheless, there is a dearth of a comprehensive coordination mechanism that encompasses all parties involved in engineering projects within its governance purview [10]. As a result, traditional research on inter-organizational conflicts has typically been confined to the relationship between the project owner and contractor. This indeed represents a blind spot in the research on inter-organizational conflict management in construction engineering. Fortunately, with the in-depth application of Social Network Theory and computer-aided simulation and modeling techniques, the trust networks structure of project participants can be depicted with relatively high accuracy [11]. This has thereby provided an effective approach for the collaborative governance of inter-organizational conflicts among numerous project participants, which is precisely the central focus of this research.

Furthermore, in the research domain of construction projects, previous research have indicated that leveraging the functional role of conflict can not only enhance team members’ recognition of tasks and the degree of mutual cooperation but also facilitate communication, discussion, and decision-making among members, thereby contributing to the improvement of project performance [12]. However, the improper utilization of conflict not only exerts a negative interference on project performance but also brings tension and disturbance to the organization. Some scholars also contend that conducting the management of inter-organizational conflict holds significant importance for achieving project performance and elevating the level of engineering management [13]. In this respect, clarifying the types of inter-organizational conflict constitutes the primary task of conflict management. Different scholars hold disparate viewpoints. Early related research emphasized that inter-organizational conflict is divided into substantive and emotional aspects, respectively referring to the inter-organizational confrontation and competition resulting from differences in work tasks and the personal emotional opposition among the participants. In reality, as a complex psychological and social phenomenon, inter-organizational conflict encompasses multiple levels and dimensions. Transformations occur among different types of conflicts. In recent years, scholars have initiated research on engineering management from the perspective of conflict management [14]. Consequently, previous related research have generally pointed out that the conflicts among the participants in construction projects encompass three aspects, namely task conflict, relationship conflict, and process conflict.

Among them, task conflict does not involve tense inter-team relationships [9]. It manifests as different opinions or viewpoints among various participants in the project implementation. Relationship conflict reflects the incompatibility of emotions among the participants, manifested as confrontational behaviors or distrust among teams. Moreover, process conflict reflects the differences among various participants in the project implementation regarding the task implementation process and the sequence of process connections [10]. Generally, it is believed that reasonable task conflict is beneficial to promoting the efficiency of cooperation among teams, while conflicts related to processes and relationships will have a negative association with the cooperation process.

### Project performance is affected by complex factors

Construction projects generally have the characteristics of one-time, constraints, systematic and holistic, making the project performance management also has its uniqueness, specifically construction projects have stronger goal orientation, management work integration, team member mobility, stakeholder relevance and so on is also an important characterisation of project performance [11]. Construction projects as an interactive system, the project participants and their performance is bound to be affected by the subjective or objective environment, the different stages of the project life cycle of the dominant factors affecting the project performance there are obvious differences, and this common factor bias and special factor bias state is unstable, dynamic [15]. Further, construction project performance management has more objectives, more stakeholders, and more complex evaluation level dimensions. Previous research have focused on the overall project revenue indicators, focusing on project cost and revenue, project progress, quality and safety, human resource management and other indicators, but for some short duration, small staff and high mobility projects, there are greater difficulties in maintaining the continuity and consistency of project performance management [16].

In fact, the performance of construction project is the classic perspective and measurement method to judge the success or failure of the project [17]. In this regard, the three aspects of cost, quality and duration are the main ways of examining the performance of construction projects, which is also applicable to construction projects. Although some research have expanded the content of project performance on the basis of the above three indicators, these three aspects are still the most universal project performance evaluation indicators. In addition, project participants’ satisfaction constitutes an indispensable part of performance as well [18]. This research covers both to represent construction projects performance. That is, the traditional three aspects and participant satisfaction are examined simultaneously.

### Model development and hypotheses

**Fig 1:**
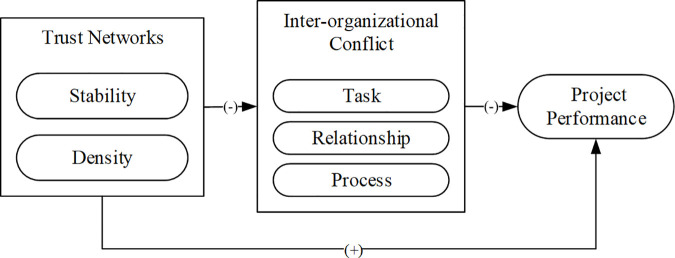
Conceptual model.

Through the theoretical background, it can be confirmed that the trust relationship between project participants does constitute a networked structure, and this trust network can affect inter-organizational conflict and project performance, but the extent and manner of their association remain unclear. At the same time, in the specific context of construction projects, the trust network between participants representing different interests can facilitate there duction of inter-organizational conflicts among them. Therefore, this research presents a conceptual model, as shown in Fig 1, to explore the interaction mechanism between trust networks, inter-organizational conflict, and project performance.

### Trust networks and inter-organizational conflict

Previous research have acknowledged that the key antecedents of conflict mainly comprise: project characteristics, formal governance mechanisms, informal governance mechanisms, network structure positions, and individual factors [19]. Among them, the informal governance mechanism emphasizes that trust relationships exert an inhibitory effect on inter-organizational conflict. Nevertheless, when considering all conflict events during the cooperation period of construction project participants as an entire research object, the traditional binary analysis perspective between the owner and contractor appears slightly insufficient in explanatory power [3]. In fact, inter-organizational conflict may occur among any two or more participants. Hence, the trust network structure from the perspective of social network theory analysis will serve as an informal governance mechanism and play a distinctive role in addressing inter-organizational conflict. From the perspective of social networks, each stakeholder who trusts another is willing to believe that the partner will not take advantage of others [5]. This is consistent with the basic concept of trust, in that one party in the binary relationship believes that the other party will not perform actions harmful to them, even if they can do so.

Consequently, when the density of the trust network is relatively dense, a relatively high-level mutual trust relationship is established among the engineering participants, thereby being capable of suppressing the degree of inter-organizational conflict from a binary perspective [14]. Then, when the degree of conflict among all the participants is significantly reduced, it indicates that all conflict events during the cooperation period of the participants have been mitigated to a certain extent. On the other hand, the stability of the trust network among the various participants of the engineering project represents the general tenacity of the trust relationship links, that is, to what extent the trust relationship among the participants can be maintained without rupture [20]. Thus, when the stability of the trust network is relatively high, it can continuously suppress the inter-organizational conflicts among the participants throughout the entire cooperation process, which implies that all conflict events during the cooperation period of the participants have been mitigated to a certain extent [21]. Thus, this research has the following hypothesis:

**H1a:** Trust networks density is negatively related to inter-organizational task conflict.

**H1b:** Trust networks density is negatively correlated with inter-organizational relationship conflict.

**H1c:** Trust networks density is negatively correlated with inter-organizational process conflict.

**H1d:** Trust networks stability is negatively related to inter-organizational task conflict.

**H1e:** Trust networks stability is negatively correlated with inter-organizational relationship conflict.

**H1f:** Trust networks stability is negatively related to inter-organizational process conflict.

### Inter-organizational conflict and project performance

From a macroscopic viewpoint, extreme or intense inter-organizational conflicts would result in the dispersion of resources, the disruption of organizational structure and order, and the reduction of group efficiency [22]. Conversely, a moderate level of inter-organizational conflicts can facilitate the full interaction of information, the simplification of business processes, the enhancement of cooperation efficiency, and the improvement of cooperation performance. Hence, outstanding project managers do not attempt to completely eliminate inter-organizational conflicts but rather endeavor to prevent highly intense conflict events from consuming excessive energy of the participating parties. During the entire life cycle of engineering construction projects, different types of conflict events will give rise to heterogeneous outcomes and exert differentiated correlations with project performance [23]. This research classifies the inter-organizational conflicts in construction projects into task conflicts, process conflicts, and relationship conflicts, and takes this as the starting point to explore their differentiated correlation with project performance.

Relational conflicts can have a considerable negative correlation with the performance and decision-making of project teams, thereby disrupting project performance. The reasons lie in the following: on the one hand, relationship conflict causes each participating party to hide their own viewpoints and opinions, restricting the flow of information [24]. On the other hand, it leads to negative emotions such as anger or hostility among the participating parties, adversely affecting the personal emotions of each member within the team. All in all, relationship conflict impedes the information interaction among the project participants, causing their focus to deviate from the construction content and tasks themselves, resulting in a decline in project performance. Moreover, process conflict is prone to emerge during the arrangement of processes and tasks. The causes include unequal status of project participants, incomplete contracts, information asymmetry, etc. Previous research have indicated that process conflict not only reduces team cohesion and affects the achievement of project performance but also leads to a decrease in the smoothness of cooperation among the participating parties, making it challenging for various teams to effectively collaborate to accomplish the tasks. Similarly, task conflict occurs when the participating parties have different opinions or disputes regarding the specific tasks of the project [25]. It is generally believed that task conflict can stimulate more creativity among the participating parties and improve the communication relationships among teams. However, it possesses a high degree of uncertainty. Therefore, the occurrence of task conflict events often exceeds expectations, requiring more time to resolve these conflict events. This easily leads to risks of project delay or cost overrun, effectively reducing the level of project performance [26]. Thus, this research has the following hypothesis:

**H2a:** Inter-organizational task conflict is negatively related to project performance.

**H2b:** Inter-organizational relationship conflict is negatively related to project performance.

**H2c:** Inter-organizational process conflict is negatively related to project performance.

### Trust networks and project performance

Previous research have partially confirmed the conclusion that the trust relationship among project participants is associated with project performance [27]. But, research exploring trust relationships from the dichotomised perspective of the developer-contractor have been dominant, where trust relationships are often seen as a complementary means or an adjunct to the contractual governance mechanism of the project [28]. However, the performance of a construction project is objectively facilitated by the joint efforts of many project participants. That is, not all project participants have a contractual relationship or affiliation with each other, thus trust relationship becomes a project governance tool that works independently beyond the contract, and may be able to have a special relationship with project performance, for which there is currently a gap in previous research [29]. This research therefore seeks to analyse the relationship between trust relationships and project performance from the perspective of network structure [30].

Indeed, social network theory has clearly pointed out that the structure of a network can interfere with the behavior of its participants, and that the behaviour of individual participants inevitably affects the performance of the network [31]. However, as the characteristic indicators of network structure are obviously heterogeneous, the degree of correlation between different characteristic indicators and the behaviors of project participants as well as project performance remains unclear [32]. In this regard, network density and relationship stability are mutually distinct yet representative indicators, which are chosen in this research to demonstrate the structural characteristics of the trust networks. Thus, this research has the following hypothesis:

**H3a:** Trust networks density is positively related to project performance.

**H3b:** Trust networks stability is positively related to project performance.

## Methods

### Questionnaire design

Questionnaire survey method was used to collect data, and the items of the survey scale were all from mature items of previous similar research [33]. In order to improve the questionnaire items, six experts were invited to discuss. They represent the government, academia, engineering consulting and design companies respectively. All of them have a master’s degree or above, and have participated in many construction projects with many years of work experience. According to the feedback from the experts, we revised the initial measurement to improve the readability and validity of the questionnaire. In addition, a clear explanation and definition of trust relationship were added to the scale for respondents to understand [34]. All the measurement items were measured on the 5-point Likert scale (1 = strongly disagree; 5 = strongly agree) and Table 1 presents the items.

**Table 1:** Items of measurement and reliability and validity analysis.

Construct	Variable	Mmeasurement items	VIF	Loading	Cronbach’s α	CR	AVE	R² values
**Trust** **Networks [35]**	Density (ND)	ND1: There is a general relationship of mutual trust between the various parties involved in the project.	3.817	0.916	0.912	0.938	0.792	-
		ND2: The participants have always tried to show that they are to be trusted.	2.940	0.885				
		ND3: All parties involved agreed that the relationship of trust had a significant impact on the project.	2.765	0.871				
		ND4: Project information is often shared efficiently, and trusting relationships emerge between the participants.	3.288	0.887				
	Stability (NS)	NS1: Relationship of trust between the participants involved in the project persists throughout the co-operation period	1.794	0.849	0.844	0.906	0.763	
		NS2:The project participants are satisfied with the trust that they have established during the project	2.121	0.867				
		NS3:The project participants want to maintain a long-term trusting relationship after the project	2.423	0.903				
**Inter-organizational** **Conflict [36]**	Task conflict (TC)	TC1: The participants have conflicting working styles and perspectives.	5.222	0.934	0.937	0.955	0.841	0.406
		TC2: There are often disagreements between the participants on the work content and interface division.	3.334	0.913				
		TC3: There are often great conflicts and dissatisfaction between the participants in the work tasks.	4.813	0.927				
		TC4: There is often disagreement among the participants about the distribution of work tasks.	2.803	0.894				
	Relationship conflict (RC)	RC1: There were many disputes in the process of communication between the participants.	2.945	0.932	0.859	0.914	0.779	0.411
		RC2: There was a clear breakdown in the relationship between the participants.	2.447	0.900				
		RC3: There was often anger and bickering among the participants.	1.846	0.812				
	Process conflict (PC)	PC1: There are differences of opinion among the participants about the way the work should be done.	2.326	0.853	0.900	0.930	0.769	0.157
		PC2: The participants have different opinions on the schedule of the construction period.	3.431	0.931				
		PC3: The participating parties often shirk their responsibilities.	2.426	0.845				
		PC4: The participating parties have different opinions on the allocation of construction resources.	2.458	0.875				
**Project** **Performance [37]** **(PP)**		PP1: The project quality accords with the standard.	3.076	0.888	0.937	0.955	0.840	0.282
		PP2: The project has come in on budget.	5.009	0.944				
		PP3: The project has come in on schedule.	3.185	0.895				
		PP4:The client is satisfied with the project outcomes.	4.627	0.938				

### Sampling and procedure

Snowball sampling method was used in this research. Previous research have shown that the core participants of construction projects mainly include various government departments, engineering design and management consulting enterprises, financial enterprises, private investment and construction enterprises. Frequent work communication and inter-organizational conflicts between them are inevitable. Based on this fact, we asked 14 engineering directors involved in major infrastructure or public facilities projects to send questionnaires to the participants of the projects they had worked on that they thought were competent. The researcher will explain to the respondents through the questionnaire that this research is only for academic purposes, and the information they fill in will be kept confidential. They will then be asked to continue to invite other participants who are qualified to fill out questionnaires for this research objective.

A total of 308 questionnaires were collected from May to December 2024. Respondents need to confirm that they have participated in the development, construction or operation process of construction project, so as to ensure that they can understand the meaning of each item in the scale and give an exact answer. The forms of the questionnaire were divided into electronic and paper version. After eliminating the questionnaires with incomplete answers, obvious arrangement of answers or contradictory answers, 207 valid questionnaires were obtained, and the effective recovery rate was 67.21%. Table 2 presents the characteristics of the respondents.

**Table 2:** Characteristics of respondents.

Background characteristics	Frequency	Percentage
Enterprise type		
Representative of the host government	56	27.05%
Local suppliers of materials or equipment	48	23.19%
Local financing support agencies	29	14.01%
Local third-party consulting agency	43	20.77%
Local contractors or subcontractors	23	11.11%
Others	8	3.86%
Work experience		
<5 years	101	48.79%
5–10 years	73	35.27%
>10 years	33	15.94%
Job position		
Manager of the headquarters	38	18.36%
Project/department manager	61	29.47%
General management/technical personnel	108	52.17%
Education		
Bachelor’s degree	87	42.03%
Master’s degree or above	44	21.26%
Others	76	36.71%

Given that all the data were collected from respondents in the construction engineering industry using the same survey questionnaire, to address the issue of common method bias, this research initially employed procedural control measures to regulate the data collection process. Specifically, a method of cross-arranging the measurement items of variables was adopted. This method aims to prevent respondents from being disturbed by guesses about the research purpose, thereby ensuring the quality of the questionnaire responses. Moreover, Harman’s single-factor test is extensively utilized in the social sciences to detect common method bias.

In this research, by utilizing the SPSS 24 analytical software, it was revealed that the variance explained by the first factor of the scale data was less than 40%, which met the threshold requirements, as presented in Table 3. Meanwhile, the data analysis demonstrated that there were six principal components, precisely corresponding to the number of variables in this research.

**Table 3:** Total variance explanation.

Principal Component	Initial eigenvalue			Determine the sum of squared loads		
	Total	Variance percentage	Accumulate %	Total	Variance percentage	Accumulate %
1	8.520	38.727	38.727	8.520	38.727	38.727
2	3.318	15.084	53.811	3.318	15.084	53.811
3	2.373	10.788	64.598	2.373	10.788	64.598
4	1.526	6.938	71.537	1.526	6.938	71.537
5	1.150	5.225	76.762	1.150	5.225	76.762
6	1.025	4.660	81.422	1.025	4.660	81.422

Overall, considering the above analysis, the common method variance issue has little interference with the validity of this research [38]. Thus, the scale sample data collected through the questionnaire can be used for subsequent analysis.

### Construct reliability and validity

In this research, the Kaiser - Meyer - Olkin (KMO) measure and Bartlett’s test of sphericity were utilized to evaluate the suitability of the scale for factor analysis. Specifically, when the KMO value exceeds 0.7 and the significance probability of the Bartlett’s test of sphericity statistic is less than or equal to the significance level, it is appropriate to proceed with the subsequent factor analysis [39]. As shown in Table 1, it can be considered that the scale has good reliability. Then factor analysis of KMO value and Barlett spherical test index data was carried out by SPSS 24 software, and the results showed that: KMO = 0.894, Approximate chi-square value = 3700.692, df = 231, Sig = 0.000, Cumulative variance of interpretation = 81.42%. This indicates that the data obtained in this research are suitable for factor analysis [40].

SmartPLS 4 software was used to analyze the valid data collected by the scale. The variables shown in Table 1 and Table 4 are validated. It can be considered that the scale used in this research has good reliability, and the obtained survey data have good validity [41].

**Table 4:** Correlation matrix and the square root of AVE of factors.

Items	ND	NS	PC	PP	RC	TC
**ND**	**0.890**					
**NS**	0.450	**0.873**				
**PC**	−0.394	−0.218	**0.877**			
**PP**	0.428	0.177	−0.213	**0.917**		
**RC**	−0.634	−0.368	0.333	−0.502	**0.883**	
**TC**	−0.494	−0.582	0.382	−0.149	0.357	**0.917**

Bold values on the diagonal represent the square root of AVE.

### Test of the structural model and hypotheses

This research utilizes the Structural Equation Model (SEM) method to validate the correlational relationships and path coefficients among variables within the theoretical conceptual model, including trust networks, inter-organizational conflicts, and project performance. As a highly prevalent statistical analysis approach in the realm of social sciences, SEM is a statistical methodology that uses a system of linear equations to depict the relationships between observed variables and latent variables, as well as among latent variables themselves. Distinct from traditional regression analysis, SEM allows researchers to concurrently examine a set of regression equations. Moreover, these equations vary significantly from traditional regression analysis in aspects such as model structure, variable configuration, and equation assumptions. That is to say, the SEM method is more diverse and inclusive in its application compared to traditional regression analysis methods, which constitutes the primary rationale for adopting this method in this research.

In actuality, the structural equation model analysis method encompasses two major mainstream technical forms: the covariance – based structural equation model (CB – SEM) and the partial least squares – based structural equation model (PLS – SEM). Specifically, the PLS-SEM method neither demands large – scale sample data nor requires the data to follow a normal distribution. More crucially, the PLS-SEM method exhibits higher statistical efficiency. These characteristics are highly congruent with the actual circumstances of the data collected in this research. Consequently, PLS – SEM is employed as the empirical analysis tool for this research [42].

It is worth noting that this research examined the issue of multicollinearity through the variance inflation factor (VIF). The results of the data analysis indicated that the VIF values of all variables were close to or below 3, suggesting that there was no problem of multicollinearity. Besides, global goodness-of-fit ($\text{GoF }=\sqrt{\overset{―}{Communality}\times \overset{―}{R^{2}}}$) is animportant index to measure the goodness of fit of PLS-SEM. After calculation, the GoF value in this research was 0.43, greater than the optimal standard value (GoF = 0.36), indicating that the model has a good goodness of fit standard.

The above-mentioned tests indicate that the data have good reliability and validity, SmartPLS 4 software was used to carry out the analysis of Bootstrapping method. The samples were 5000 times, recommended by previous research findings. The results of testing are shown in Table 5, where P value represents the level of significance. In general, the smaller the P value, the better the significance. Therefore, it can be judged that some hypotheses are not supported by data.

**Table 5:** Results of hypotheses testing.

Path	Standard path coefficient	T-statistics	P value	Correlation	Hypothesis	Inference
**ND - > TC**	−0.291	3.8	0.000***	Negative	H1a	Supported
**ND - > RC**	−0.588	10.358	0.000***	Negative	H1b	Supported
**ND - > PC**	−0.371	5.84	0.000***	Negative	H1c	Supported
**NS - > TC**	−0.451	6.516	0.000***	Negative	H1d	Supported
**NS - > RC**	−0.103	1.547	0.122 N.S.	/	H1e	/
**NS - > PC**	−0.051	0.766	0.444 N.S.	/	H1f	/
**TC - > PP**	0.108	1.501	0.133 N.S.	/	H2a	/
**RC - > PP**	−0.390	5.377	0.000***	Negative	H2b	Supported
**PC - > PP**	−0.038	0.544	0.586 N.S.	/	H2c	/
**ND - > PP**	0.224	3.019	0.003**	Positive	H3a	Supported
**NS - > PP**	−0.013	0.174	0.862 N.S.	/	H3b	/

Note: * means P < 0.05; ** means P < 0.01; *** means P < 0.001; N.S. means Nonsignificant.

Based on the above analysis, it can be inferred that the correlation between the trust networks and project performance may be indirectly affected by inter-organizational conflicts. Consequently, the above-mentioned steps of the Bootstrapping calculation were replicated (with a minimum of 5,000 samples). The analysis results of the indirect correlation path are shown in Table 6. Specifically, conflicts among organizations do indeed have an indirect association with the trust networks and project performance. Although this indirect association was not anticipated during the initial formation of theoretical hypotheses, this analysis outcome suggests that relevant project governance strategies can be concurrently implemented for both the trust networks and inter-organizational conflicts. These two aspects can collaborate to enhance project performance.

**Table 6:** Results of indirect association test.

Indirect Path	Standard path coefficient	T-statistics	P value
**ND - > PC - > PP**	0.014	0.518	0.604 N.S.
**NS - > PC - > PP**	0.002	0.285	0.775 N.S.
**ND - > TC - > PP**	−0.031	1.251	0.211 N.S.
**ND - > RC - > PP**	0.23	4.76	0.000 ***
**NS - > TC - > PP**	−0.049	1.422	0.155 N.S.
**NS - > RC - > PP**	0.04	1.544	0.123 N.S.

Note: * means P < 0.05; ** means P < 0.01; *** means P < 0.001; N.S. means Nonsignificant.

## Results and discussion

### Trust networks can inhibit inter-organizational conflicts

**Fig 2:**
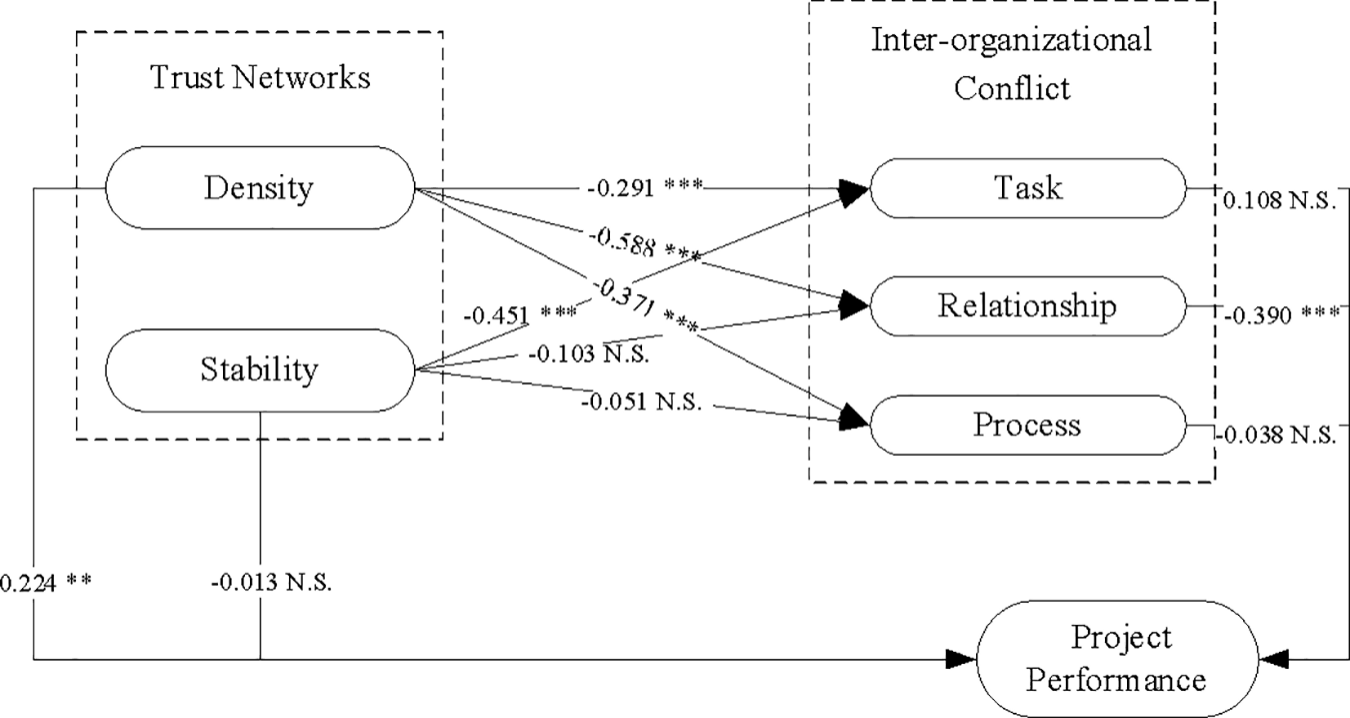
Structural model diagram with path coefficients. Note: * means P < 0.05; ** means P < 0.01; *** means P < 0.001; N.S. means Nonsignificant.

According to the data analysis results of this research (Fig 2), hypotheses H1a to H1d are all supported. This research outcome demonstrates that the presence of a trust networks structure can inhibit three common types of inter-organizational conflict events. Put differently, if the density of the trust networks among the participants in an engineering project is low or its stability is poor, it is highly probable to result in an escalation of inter-organizational conflicts among the participants. Nevertheless, hypotheses H1e and H1f did not receive empirical support. Specifically, the stability of the trust networks failed to exhibit a clear correlation with inter-organizational relationship conflict and process conflict. This further suggests that the impact of the stability of the trust networks structure on inter-organizational conflict is not all-encompassing. Notwithstanding, as hypothesis H1d was validated by the data, indicating a distinct negative correlation between the stability of the trust networks and inter-organizational task conflict, it is thus inaccurate to claim that the stability of the trust networks structure has no bearing on inter-organizational conflict.

In the traditional binary trust relationship research perspective, trust, as an informal project governance approach, it is related to conflicts among organizations, and previous research have shown an inverted U-shaped relationship between the two. Nevertheless, when the research perspective enters the trust networks structure, the analytical results of this research present a more distinctive conclusion, namely, the existence of a trust networks can significantly suppress inter-organizational conflicts among the participants of an engineering project, which is conducive to enhancing the organizational management model and project governance scheme of engineering projects.

Overall, a substantial body of prior research has acknowledged the significant implications of informal governance mechanisms, exemplified by trust relationships, in construction projects. However, trust relationships are not merely a dichotomy between the owner and contractor. Instead, numerous project participants should be taken into consideration. Consequently, the data analysis findings of this research present a rather significant breakthrough. That is, project governance strategies regarding the density and stability of the trust network should be considered holistically, rather than simply focusing on enhancing the trust level between the owner and contractor.

### Inter-organizational relationship conflict hinders project performance

The findings indicated that Hypothesis H2b was supported (Fig 2). This implies a negative correlation between inter-organizational relationship conflict and project performance. This represents the pivotal academic breakthrough attained in this research. Conversely, Hypotheses H2a and H2c did not receive support from the data analysis outcomes. These results from the correlation analysis suggest that not all manifestations of inter-organizational conflict impede project performance. This research conclusion is derived from the perspective of analyzing the diversified network structure, which bears similarities to the conclusions drawn from the binary analysis perspective of project participants in previous research.

For example, in prior studies, process conflicts between the project owner and the contractor were likely to enhance project performance since such conflicts facilitated the efficiency of information interaction. However, from the viewpoint of the relationship network among participants, process conflicts among two or more parties frequently give rise to practical dilemmas, such as evading responsibilities, shifting blame, and creating obstacles for one another. Consequently, all project participants are compelled to allocate additional costs or time to resolve the process conflicts. Thus, from the multifaceted perspective of numerous participants, whether inter-organizational process conflicts essentially impede or boost project performance remains a topic that warrants further investigation. Based on the current data results, a conclusive and consistent answer cannot be obtained.

### Correlation between trust networks and project performance is inconsistent

The results show that hypotheses H3a is supported. But hypotheses H3b is not supported by the data. In fact, previous research have pointed out that trust networks have heterogeneous associations with the performance of engineering projects, but the data analysis results of this research show inconsistent conclusions with previous research. This difference occurs in terms of network stability (H3b). The data analysis results of this research show that there is no obvious correlation between the stability of the trust networks and the project performance. The main reason for this analysis result is that the data in this research came from infrastructure project participants with different duration of participation in the project and social status, most of whom did not participate in the whole construction process, so the trust networks is obviously in a relatively unstable dynamic change, and a more consistent conclusion cannot be drawn based on this. In previous research within this domain, little attention has been paid to the association among the length of time spent participating in projects, social status, and the stability of trust relationships, which also provides a direction for future in-depth research.

Encouragingly, when considering the indirect association of inter-organizational conflicts, a clear governance pathway can be delineated (Table 6). Specifically, when the governance strategy of enhancing the density of the trust networks while reducing inter-organizational relationship conflicts is implemented concurrently, project performance will be remarkably enhanced. This represents a breakthrough discovery that sets it apart from previous research.

### Implications

During the implementation process of construction projects, systematic management plans or work guidelines are requisite. Such documented institutional arrangements frequently emerge in the appendices or supplementary agreements of the formal contracts concluded between the owner and the contractor, subcontractors, suppliers, or other participating parties. They typically encompass detailed stipulations on work responsibilities, work procedures, risk allocation, etc., which are so voluminous that they cannot be fully encompassed in the main body of the contract. Specifically, detailed institutional arrangements regarding the process of project variations, the effective elements of project visas, special construction plans, and communication channels among project participants will be presented in the form of appendices or supplementary agreements as supplements to the formal contract. All participating parties of the project have committed to adhering to these supplementary agreements upon the signing of the formal contract. The trust networks structure among project participants emphasized in this research often stems from the initial trust relationships among them. Nevertheless, when the project enters the implementation stage, the persistence, reinforcement, or disruption of trust relationship links is closely associated with the aforementioned systematic management plans or work guidelines.

In accordance with the analytical conclusion of this research, the density of the trust networks is capable of inhibiting multiple forms of inter-organizational conflicts and concurrently facilitating project performance. Correspondingly, the stability of the trust networks constitutes a relatively independent influencing factor. It does not directly promote the achievement of project performance, but it can suppress inter-organizational task conflicts among project participants. Hence, it is of paramount importance to promote the enhancement of trust relationships among all project participants throughout the entire project process through rational institutional arrangements. The greater the number of trust relationship links among project participants, the better the density and stability of the trust networks. Consequently, inter-organizational conflicts will be mitigated, and project performance will be effectively elevated. For instance, Building Information Modeling (BIM) technology can be employed for digital twin modeling of engineering projects. On this basis, the information and resource sharing among project participants can be facilitated, while key data such as project cost, project progress, and quality can be updated in real time. Simultaneously, the breaches of contract or illegal and non-compliant behaviors of project participants can be disclosed. Through multiple means, a favorable cooperative ambiance among project participants can be formed to ensure that the trust networks formed by project participants maintains a high level of density and stability.

Rather intriguingly, this research has derived the mechanism of the correlation between inter-organizational conflicts and project performance from multiple perspectives, which contrasts with the conclusions regarding process conflicts in previous research from a binary perspective. Then, when considering the actual situation where multiple participating parties collaborate to complete the project construction, any inter-organizational conflicts will impede project performance. To this end, establishing a systematic management plan or work guideline to mitigate various forms of inter-organizational conflicts constitutes an effective approach to enhance project performance. That is, although the contract is imperfect, detailed stipulations such as work responsibilities, work procedures, and risk allocation can be required to be jointly signed and adhered to by all participating parties in the form of supplementary agreements. Simultaneously, adopting the fundamental concept of the Integrated Project Delivery (IPD) model, with the objective of reducing inter-organizational conflicts, all participating parties are expected to jointly deliberate and formulate a systematic management plan or work guideline, rather than having the owner solely formulate a vast number of rigid contractual provisions.

## Conclusions

This research integrates a diverse range of network perspectives into the examination of inter-organizational conflicts among the participants in engineering projects. It elucidates the correlative mechanisms among trust networks, inter – organizational conflicts, and project performance among these participants, thereby bridging the research gap in the field of project governance. Unlike previous research, in the diversified analytical perspective, different forms of inter-organizational conflicts significantly impede the achievement of project performance. However, inter-organizational conflicts can be suppressed by enhancing the density of the trust networks, maintaining the stability of the network structure, and other methods, thereby attaining the goal of improving project performance. Moreover, the indirect correlation of inter-organizational conflicts has also been revealed, which has not been covered in previous research. Consequently, when formulating project management charters or regulations, project managers should integrate both trust networks and governance strategies for inter – organizational conflicts. By doing so, it is possible to comprehensively enhance project performance.

Subsequent research can further utilize methods such as computer simulation to analyze the correlation of the dynamic changing relationship network with inter-organizational conflicts and project performance. Additionally, the antecedent influencing factors of the structural characteristics of trust networks can be analyzed to clarify the core roots that dominate the trust networks structure among the construction participants of engineering projects. Informed consent was obtained from all subjects involved in the research.

## Supporting information

S1 File Questionnaire of Survey. (PDF)

S2 File Primary Data. (PDF)

## References

[1] Capaldo A, Giannoccaro I. Interdependence and network-level trust in supply chain networks: A computational study. Industrial Marketing Manag. 2015;44:180–95. doi: 10.1016/j.indmarman.2014.10.001

[2] Giannoccaro I, Iftikhar A. Is Network Trust Beneficial For Supply Network Resilience? A Simulation Analysis. IFAC-PapersOnLine. 2019;52(13):2437–42. doi: 10.1016/j.ifacol.2019.11.572

[3] Wang X, Yin Y, Deng J, Xu Z. Influence of trust networks on the cooperation efficiency of PPP projects: moderating effect of opportunistic behavior. J Asian Architect Building Engineering. 2021;22(4):2275–90. doi: 10.1080/13467581.2021.1972002

[4] Posselt JR. Trust Networks: A New Perspective on Pedigree and the Ambiguities of Admissions. Rev Higher Educ. 2018;41(4):497–521. doi: 10.1353/rhe.2018.0023

[5] Wang X, Yin Y, Deng J, Xu Z. Opportunistic Behavior Governance in PPP Projects: An Analysis Based on Trust Networks. Ad Civil Eng. 2021;2021(1). doi: 10.1155/2021/8899338

[6] Shen W, Tang W, Wang S, Duffield CF, Hui FKP, You R. Enhancing Trust-Based Interface Management in International Engineering-Procurement-Construction Projects. J Constr Eng Manage. 2017;143(9). doi: 10.1061/(asce)co.1943-7862.0001351

[7] Girmscheid G, Brockmann C. Inter- and Intraorganizational Trust in International Construction Joint Ventures. J Constr Eng Manage. 2010;136(3):353–60. doi: 10.1061/(asce)co.1943-7862.0000142

[8] Barough AS, Shoubi MV, Skardi MJE. Application of Game Theory Approach in Solving the Construction Project Conflicts. Procedia - Social and Behavioral Sciences. 2012;58:1586–93. doi: 10.1016/j.sbspro.2012.09.1145

[9] Wu G, Zhao X, Zuo J. Effects of inter-organizational conflicts on construction project added value in China. Int J Confl Manag. 2017;28(5):695–723. doi: 10.1108/ijcma-03-2017-0025

[10] Wang X, Yin Y. Research on the influencing factors of trust networks of construction project participants. Buildings. 2025;15(11):1784. doi: 10.3390/buildings15111784

[11] Lv J, Wang X, Chen Y. Agent-based simulation of trust networks and opportunistic behaviours of hydraulic infrastructure project participants. PLoS One. 2025;20(1):e0316992. doi: 10.1371/journal.pone.0316992 39761300 PMC11702997

[12] Panteli N, Sockalingam S. Trust and conflict within virtual inter-organizational alliances: a framework for facilitating knowledge sharing. Decision Support Systems. 2005;39(4):599–617. doi: 10.1016/j.dss.2004.03.003

[13] Lim BTH, Loosemore M. The effect of inter-organizational justice perceptions on organizational citizenship behaviors in construction projects. Int J Project Management. 2017;35(2):95–106. doi: 10.1016/j.ijproman.2016.10.016

[14] He Q, Luo L, Hu Y, Chan APC. Measuring the complexity of mega construction projects in China—A fuzzy analytic network process analysis. Int J Project Manag. 2015;33(3):549–63. doi: 10.1016/j.ijproman.2014.07.009

[15] Maloney WF. Framework for Analysis of Performance. J Constr Eng Manage. 1990;116(3):399–415. doi: 10.1061/(asce)0733-9364(1990)116:3(399

[16] Sun H, Tang W, Duffield CF, Zhang L, Hui FKP. How to get international construction projects delivered on time: from chinese contractors’ perspective. J Civil Eng Manag. 2022;28(2):134–49. doi: 10.3846/jcem.2022.16381

[17] Lin Y-H, Zhang H, Kim CJ, Xu Z, Xu S. The Influence of Cultural Intelligence and Institutional Distance of Chinese and Korean Contractors on the Performance of International Construction Projects. KSCE J Civil Eng. 2021;25(9):3223–34. doi: 10.1007/s12205-021-1694-1

[18] Yan L, Zhang L. Interplay of Contractual Governance and Trust in Improving Construction Project Performance: Dynamic Perspective. J Manage Eng. 2020;36(4). doi: 10.1061/(asce)me.1943-5479.0000791

[19] Jaffar N, Tharim AHA, Shuib MN. Factors of Conflict in Construction Industry: A Literature Review. Procedia Engineering. 2011;20:193–202. doi: 10.1016/j.proeng.2011.11.156

[20] Wang X, Yin Y, Xu Z. The influence of trust networks on public–private partnership project performance. Proceedings of the Institution of Civil Engineers - Management, Procurement and Law. 2024;177(2):65–74. doi: 10.1680/jmapl.21.00025

[21] Prieto-Remón TC, Cobo-Benita JR, Ortiz-Marcos I, Uruburu A. Conflict Resolution to Project Performance. Procedia - Social and Behavioral Sciences. 2015;194:155–64. doi: 10.1016/j.sbspro.2015.06.129

[22] Zhang L, Huo X. The impact of interpersonal conflict on construction project performance. International Journal of Conflict Management. 2015;26(4):479–98. doi: 10.1108/ijcma-09-2014-0072

[23] Wu G, Zhao X, Zuo J. Relationship between Project’s Added Value and the Trust–Conflict Interaction among Project Teams. J Manage Eng. 2017;33(4). doi: 10.1061/(asce)me.1943-5479.0000525

[24] Wu G, Liu C, Zhao X, Zuo J. Investigating the relationship between communication-conflict interaction and project success among construction project teams. Int J Project Manag. 2017;35(8):1466–82. doi: 10.1016/j.ijproman.2017.08.006

[25] Lee C, Won JW, Jang W, Jung W, Han SH, Kwak YH. Social conflict management framework for project viability: Case studies from Korean megaprojects. Int J Project Manag. 2017;35(8):1683–96. doi: 10.1016/j.ijproman.2017.07.011

[26] Ng HS, Peña-Mora F, Tamaki T. Dynamic Conflict Management in Large-Scale Design and Construction Projects. J Manage Eng. 2007;23(2):52–66. doi: 10.1061/(asce)0742-597x(2007)23:2(52

[27] Carbonara N, Pellegrino R. Public-private partnerships for energy efficiency projects: A win-win model to choose the energy performance contracting structure. J Cleaner Production. 2018;170:1064–75. doi: 10.1016/j.jclepro.2017.09.151

[28] Jayasuriya S, Zhang G, Yang RJ. Exploring the impact of stakeholder management strategies on managing issues in PPP projects. Int J Construction Management. 2020;20(6):666–78. doi: 10.1080/15623599.2020.1753143

[29] Choi EW, Özer Ö, Zheng Y. Network Trust and Trust Behaviors Among Executives in Supply Chain Interactions. Management Science. 2020;66(12):5823–49. doi: 10.1287/mnsc.2019.3499

[30] Xu Z, Yin Y, Li D, Browne GJ. Owner’s Risk Allocation and Contractor’s Role Behavior in a Project: A Parallel-mediation Model. Engineering Management Journal. 2018;30(1):14–23. doi: 10.1080/10429247.2017.1408388

[31] Granovetter M. Economic Action and Social Structure: The Problem of Embeddedness. Am J Sociol. 1985;91(3):481–510. doi: 10.1086/228311

[32] Weimann G. The strength of weak conversational ties in the flow of information and influence. Social Networks. 1983;5(3):245–67. doi: 10.1016/0378-8733(83)90027-8

[33] Nyuur RB, Brecic R, Debrah YA. SME international innovation and strategic adaptiveness. IMR. 2018;35(2):280–300. doi: 10.1108/imr-11-2015-0239

[34] Ye Z, Shen QTS. Structural equation modeling of the intelligent manufacturing entrepreneurship’s network characteristics. J Intelligent Fuzzy Systems. 2020;38(6):7803–11. doi: 10.3233/jifs-179850

[35] Wang X, Yin Y. Structural Dimensions and Measurement of Trust Networks among Construction Project Participants. Sustainability. 2023;15(5):4112. doi: 10.3390/su15054112

[36] Jehn KA. A Multimethod Examination of the Benefits and Detriments of Intragroup Conflict. Administr Sci Quart. 1995;40(2):256. doi: 10.2307/2393638

[37] Zhang L, Fu Y, Lai J, Chen Y. Complements or substitutes? Recipes of contract design, contract enforcement, and trust for enhanced project performance. Int J Project Management. 2024;42(3):102587. doi: 10.1016/j.ijproman.2024.102587

[38] Podsakoff PM, MacKenzie SB, Lee J-Y, Podsakoff NP. Common method biases in behavioral research: a critical review of the literature and recommended remedies. J Appl Psychol. 2003;88(5):879–903. doi: 10.1037/0021-9010.88.5.879 14516251

[39] Hair JF, Ringle CM, Sarstedt M. PLS-SEM: Indeed a Silver Bullet. J Marketing Theory and Practice. 2011;19(2):139–52. doi: 10.2753/mtp1069-6679190202

[40] Astrachan CB, Patel VK, Wanzenried G. A comparative study of CB-SEM and PLS-SEM for theory development in family firm research. J Family Business Strategy. 2014;5(1):116–28. doi: 10.1016/j.jfbs.2013.12.002

[41] Yan S, Liu N, Chen M, Liu Y, Han S. The thermal effect of the tandem kang model for rural houses in Northern China: a case study in Tangshan. J Asian Architecture and Building Eng. 2020;21(2):187–96. doi: 10.1080/13467581.2020.1839468

[42] Leguina A. A primer on partial least squares structural equation modeling (PLS-SEM). Int J Res Method Educ. 2015;38(2):220–1. doi: 10.1080/1743727x.2015.1005806

