# Supplementary material for: Inter-organizational conflict and construction project performance: Influencing mechanism based on trust networks
Source: PLoS One. 2025 Aug 29;20(8):e0331014. doi: 10.1371/journal.pone.0331014 (PMC12396670; doi:10.1371/journal.pone.0331014)
Supplement: S1 File — (PDF) [file pone.0331014.s001.pdf]

Questionnaire of Survey

Dear Respondents,

Hello! Thank you very much for taking time out of your busy schedule to fill out this questionnaire!

This research was carried out in strict compliance with the Declaration of Helsinki and all applicable ethical regulations. Prior to filling out the survey questionnaire, all participating respondents were explicitly informed that submitting the questionnaire was tantamount to their signing a written informed consent form. In the event that a respondent did not consent to participate in this research or the relevant researcher failed to obtain the respondent's informed consent, the respondent should refrain from submitting the questionnaire or answering the items therein.

The research team hereby promises that all the information you fill in will be kept strictly confidential and used for academic research only. The returned questionnaires will be statistically processed according to strict procedures and will not refer to specific units or individuals. Thank you again for your support! All questions in this questionnaire will not involve your work confidentiality and personal privacy, only need to answer in detail according to the real situation and your work experience.

In practice, there are many participants in construction projects, and the relationship of trust or distrust among them forms a network structure. The purpose of this questionnaire is to explore the influence of the trust network structure of the participants in transnational projects on the cooperation process.

May 2024

Basic information of the anonymous respondents

(Note: Please select the most appropriate option according to your actual situation and tick " √ " in the box of the corresponding option. Each item is a single choice.)

**Background characteristics**	**Options (√)**
Enterprise type	
Representative of the host government	
Local suppliers of materials or equipment	
Local financing support agencies	
Local third-party consulting agency	
Local contractors or subcontractors	
Others	
Work experience	

<5 years	
5–10 years	
>10 years	
Job position	
Manager of the headquarters	
Project/department manager	
General management/technical personnel	
Education	
Bachelor's degree	
Master's degree or above	
Others	

Information collection for sample items

(Note: Please recall the construction stage of engineering projects you have participated in, select the most impressive and familiar project, and fill in the following questions based on this, tick " √ " in the corresponding box. Each item is a single choice.)

Item and quantitative index (score)		Strongly disagree	Slightly disagree	Neutrality	Slightly agree	Strongly agree
Item		1	2	3	4	5
Trust Networks	There is a general relationship of mutual trust between the various parties involved in the project.					
	The participants have always tried to show that they are to be trusted.					
	All parties involved agreed that the relationship of trust had a significant impact on the project.					
	Project information is often shared efficiently, and trusting relationships emerge between the participants.					
	Relationship of trust between the participants involved in the project persists throughout the co-operation period					
	The project participants are satisfied with the trust that they have established during the project					
	The project participants want to maintain a long-term trusting relationship after the project					

Inter-organizational Conflict	The participants have conflicting working styles and perspectives.					
	There are often disagreements between the participants on the work content and interface division.					
	There are often great conflicts and dissatisfaction between the participants in the work tasks.					
	There is often disagreement among the participants about the distribution of work tasks.					
	There were many disputes in the process of communication between the participants.					
	There was a clear breakdown in the relationship between the participants.					
	There was often anger and bickering among the participants.					
	There are differences of opinion among the participants about the way the work should be done.					
	The participants have different opinions on the schedule of the construction period.					
	The participating parties often shirk their responsibilities.					
	The participating parties have different opinions on the allocation of construction resources.					
Project Performance	The project quality accords with the standard.					
	The project has come in on budget.					
	The project has come in on schedule.					
	The client is satisfied with the project outcomes.					

This is the end of the questionnaire, please carefully check whether there are any missing questions! If you have any comments or suggestions on this questionnaire, please fill in the line below:

____
