# Supplementary material for: Inter-organizational conflict and construction project performance: Influencing mechanism based on trust networks
Source: PLoS One. 2025 Aug 29;20(8):e0331014. doi: 10.1371/journal.pone.0331014 (PMC12396670; doi:10.1371/journal.pone.0331014)
Supplement: S2 File — (PDF) [file pone.0331014.s002.pdf]

ND1	ND2	ND3	ND4	NS1	NS2	NS3	TC1	TC2
1	4	1	5	3	4	4	2	1
4	1	5	1	5	4	5	1	1
5	1	1	5	4	4	5	2	1
1	5	5	1	4	5	3	2	2
1	1	1	1	4	5	3	2	1
1	1	1	1	4	4	3	1	1
1	1	1	1	3	5	5	2	2
1	1	1	1	3	4	4	1	1
1	1	1	1	5	5	3	1	2
1	1	1	1	5	4	4	2	2
3	1	1	5	1	1	1	2	2
5	1	1	5	1	4	4	1	1
4	1	1	5	3	1	1	2	2
5	1	1	5	1	1	3	2	3
5	1	3	5	4	4	4	2	2
4	5	5	5	1	5	3	1	2
4	5	5	4	3	4	3	3	2
4	5	5	4	4	1	5	2	2
4	5	4	5	1	5	3	2	2
4	5	5	4	5	1	1	1	2
4	5	5	5	1	5	4	1	1
4	4	5	5	4	1	1	2	1
5	5	3	4	4	5	5	2	1
4	4	4	4	3	4	5	2	1
4	5	5	5	4	3	3	1	1
5	5	4	5	4	4	4	5	1
5	5	5	5	3	5	3	5	1
5	4	5	4	3	3	5	5	1
5	4	3	5	4	4	3	5	5
5	5	3	5	5	5	5	5	5
5	5	5	5	3	5	4	5	5
4	4	3	5	1	1	1	5	5
4	5	3	5	1	1	1	1	3
4	5	5	5	1	1	1	1	3
4	5	3	4	1	1	1	2	3
4	5	5	5	1	1	1	2	3
4	4	3	4	1	1	1	2	3
4	3	4	4	1	1	1	2	3
5	4	3	5	5	3	3	1	3
4	4	5	3	3	5	4	1	2
5	5	4	5	4	3	4	2	1
4	5	4	5	4	3	3	2	2
3	4	5	5	5	4	4	2	2
5	5	5	5	5	5	5	1	2
5	3	5	4	4	4	5	1	1
5	4	5	4	5	4	4	2	1
5	4	4	5	3	5	3	2	1
5	4	4	4	5	5	5	2	1
5	5	5	4	3	5	5	2	2
4	5	5	5	3	5	3	1	2
5	4	4	4	4	3	4	1	2
4	5	5	4	3	5	3	1	2
4	5	4	4	3	3	4	5	5
4	5	4	3	5	4	3	5	5
5	5	5	4	3	4	3	5	5
4	5	4	3	3	3	3	5	5
5	5	4	4	3	4	5	5	5

4	4	4	5	4	4	4	5	5
5	5	5	4	4	5	4	5	5
5	5	4	4	4	3	5	5	5
1	3	2	1	2	3	2	5	5
2	2	1	1	1	2	2	5	5
3	2	1	3	1	1	2	5	5
2	2	1	2	2	3	2	5	5
2	2	3	2	2	1	1	5	5
1	2	1	2	2	3	2	4	3
3	1	2	1	2	2	2	4	3
2	3	1	2	2	2	1	4	3
3	2	2	2	2	1	2	4	3
2	1	2	1	1	3	2	3	3
2	2	3	2	2	1	1	5	3
1	1	1	1	2	2	2	3	4
1	1	1	1	2	2	2	5	4
1	1	1	1	2	2	2	5	5
1	1	1	1	2	2	2	5	4
1	1	1	1	2	2	2	5	4
1	1	1	1	2	2	2	5	5
1	1	1	1	2	2	2	5	5
1	1	1	1	2	2	2	4	5
1	1	1	1	2	2	2	5	5
1	1	1	1	2	2	2	4	4
1	1	1	1	2	2	2	4	5
1	1	1	1	2	2	2	5	4
1	1	1	1	3	3	3	4	4
1	1	1	1	3	3	3	5	4
1	1	1	1	3	3	3	4	5
1	1	1	1	3	3	3	4	4
1	1	1	1	3	3	3	4	4
1	1	1	1	3	3	3	5	4
1	1	1	1	3	3	3	5	4
1	1	1	1	3	3	3	4	5
1	1	1	1	3	3	3	4	4
3	1	2	1	3	3	3	3	3
1	1	1	2	3	3	3	3	3
2	2	3	1	3	3	3	3	3
2	1	3	3	3	3	3	3	3
1	2	1	2	3	3	3	3	3
1	3	2	1	3	3	3	3	3
2	2	1	1	3	3	3	3	3
1	2	1	2	3	3	3	3	3
2	1	1	1	3	3	3	3	3
1	3	1	3	2	3	2	3	3
2	2	1	3	1	2	2	3	3
1	3	1	2	1	1	2	3	3
3	1	1	3	2	3	2	3	3
1	3	1	3	2	2	1	3	3
2	1	2	1	2	2	1	5	5
1	2	3	3	2	1	2	5	5
1	3	2	1	1	3	1	5	5
1	2	2	3	2	1	1	5	4
1	3	2	2	1	2	2	5	5
1	2	1	1	2	2	1	5	4
2	1	2	3	1	1	1	5	4
2	3	1	2	1	2	1	5	4
2	2	1	2	1	1	1	5	4

1	1	3	2	2	1	2	5	5
3	1	1	3	1	3	2	5	5
2	2	3	3	2	3	2	5	4
1	3	3	1	2	1	2	5	4
3	2	1	2	1	1	1	5	5
1	2	3	2	1	3	2	5	4
1	2	1	3	1	1	1	5	5
1	1	2	1	2	3	1	5	5
3	2	2	3	2	3	2	5	5
2	2	1	1	2	1	2	5	4
3	3	2	3	2	2	1	5	4
1	1	3	1	2	3	2	5	5
2	1	2	3	1	2	2	5	5
3	2	2	2	2	1	1	5	5
1	1	3	3	1	2	1	5	4
1	3	2	2	2	3	2	5	5
1	1	2	3	2	2	2	5	4
2	2	1	1	2	1	2	5	4
2	2	1	3	2	3	2	5	5
1	2	1	1	1	1	1	5	5
2	1	2	3	2	2	3	5	4
1	2	1	2	1	2	2	5	5
2	2	1	3	2	3	2	5	5
1	2	2	2	1	1	1	5	4
2	2	1	2	1	1	2	5	4
1	1	2	2	2	3	2	5	4
2	2	2	2	2	2	3	5	5
2	1	2	3	3	2	1	4	4
2	3	1	1	2	1	1	4	5
3	1	1	1	1	3	1	5	4
1	2	1	2	2	1	2	5	4
2	1	2	3	1	1	2	5	5
1	3	1	1	1	3	2	4	4
2	2	1	1	2	1	1	4	4
3	3	2	3	2	1	2	4	5
1	2	2	1	2	2	2	3	3
2	3	1	3	2	1	1	4	3
1	2	1	2	1	3	2	4	3
2	1	1	3	2	3	2	5	3
1	2	1	1	2	1	2	2	3
5	5	3	4	4	5	3	2	3
5	4	1	4	3	3	4	2	2
4	5	1	4	5	4	5	2	2
4	4	4	4	3	5	4	3	2
4	4	4	4	4	3	3	3	1
4	5	4	4	4	5	1	3	1
5	4	4	5	4	3	4	1	1
5	4	5	4	3	4	3	1	1
4	5	5	4	4	3	4	3	1
4	5	4	5	5	5	5	2	1
2	2	2	2	5	3	4	2	1
2	2	2	2	3	4	3	3	1
2	2	2	2	4	4	5	2	1
2	2	2	2	5	5	5	1	1
2	2	2	2	1	3	4	3	3
2	2	2	2	3	3	4	1	1
2	2	2	2	3	3	3	2	2
2	2	2	2	3	5	5	1	1

2	2	2	2	4	4	3	2	2
2	2	2	2	4	4	4	2	1
2	2	2	2	5	3	4	1	2
2	2	2	2	4	3	5	1	1
2	2	2	2	3	5	4	3	2
2	2	2	2	4	5	3	1	2
4	4	4	5	3	5	4	2	1
5	4	4	5	4	4	4	2	1
1	1	2	2	2	2	1	3	3
2	1	1	2	2	3	3	4	5
1	2	3	3	2	3	3	4	4
1	2	1	1	1	4	5	4	4
1	2	2	1	2	4	5	5	3
2	1	1	3	2	3	3	4	3
2	3	2	2	2	5	4	2	4
3	1	1	4	1	2	4	3	2
2	3	1	3	2	1	1	4	4
1	2	1	2	1	3	2	4	4
2	1	1	3	2	3	2	5	5
1	2	1	1	2	1	2	2	3
5	5	3	4	4	5	3	2	1
5	4	3	4	3	3	4	2	1
4	5	3	4	5	4	5	2	1
4	5	4	3	3	3	3	2	1
5	5	4	4	3	4	5	3	1
4	4	4	5	4	4	4	1	1
5	5	5	4	4	5	4	2	1
5	5	4	4	4	3	5	2	1
1	3	2	1	2	3	2	3	4
2	2	1	1	1	2	2	4	5
3	2	1	3	1	1	2	3	5
2	1	1	2	2	3	2	4	3
2	1	3	2	2	1	1	3	3
1	1	1	2	2	3	2	4	4

TC3	TC4	RC1	RC2	RC3	PC1	PC2	PC3	PC4
1	2	5	5	5	3	3	4	3
2	2	5	5	5	4	4	4	3
1	1	5	5	5	4	4	4	3
2	2	5	5	5	5	5	4	3
2	2	5	5	5	2	3	4	3
1	1	5	5	5	2	1	2	2
2	1	5	5	5	2	2	1	2
1	2	5	5	5	2	2	2	1
1	1	5	5	5	3	2	2	3
1	2	5	5	5	3	1	2	3
2	2	5	5	5	3	1	1	3
2	2	5	5	5	1	1	2	1
1	1	5	5	5	1	2	2	2
1	2	5	5	5	1	2	1	3
2	2	2	2	2	1	2	2	2
1	2	3	2	1	2	1	3	1
2	2	2	2	2	3	2	1	2
2	1	1	1	2	2	1	3	2
2	2	2	2	3	2	1	3	3
2	2	2	1	3	2	1	1	3
3	2	2	1	3	1	1	1	3
2	1	2	2	2	1	2	1	1
2	2	1	1	3	2	1	2	1
2	1	2	2	2	3	1	3	1
1	1	2	2	2	3	3	4	3
5	2	2	2	3	4	4	4	3
5	2	2	1	3	4	4	4	3
5	2	1	2	3	5	5	4	3
5	1	2	2	2	2	3	4	3
5	2	1	1	2	3	1	2	1
5	1	1	2	3	2	2	1	2
5	2	1	1	2	1	1	1	2
2	1	1	1	3	3	1	3	1
1	1	2	2	3	2	1	2	2
1	2	1	1	2	1	1	3	1
1	2	2	1	3	2	1	3	1
1	1	2	2	3	3	1	1	2
1	2	2	2	2	3	2	2	2
2	1	2	1	2	3	2	3	2
2	2	2	1	3	2	1	2	2
1	2	2	1	2	2	2	3	2
2	1	1	2	3	3	3	4	3
2	1	2	1	3	4	4	4	3
1	2	2	2	3	4	4	4	3
2	1	2	1	2	5	5	4	3
1	2	2	2	3	2	3	4	3
1	2	2	3	2	2	2	2	2
1	1	1	3	2	3	2	2	2
1	1	3	1	3	2	1	2	3
1	2	2	2	1	1	2	3	2
1	2	2	3	3	3	2	2	1
2	2	3	2	2	3	1	3	2
5	5	3	3	3	2	2	2	3
5	5	2	2	3	2	2	3	2
5	5	4	2	2	2	2	4	2
5	5	2	1	3	3	2	4	1
5	5	2	2	2	2	1	1	1

5	5	1	1	2	2	1	1	2
5	5	2	3	2	3	2	1	1
5	5	1	2	2	3	2	3	1
5	5	5	3	5	4	4	5	4
5	5	3	3	5	3	5	4	5
5	5	3	5	5	4	4	5	5
5	5	5	5	5	4	3	5	3
5	5	5	3	2	3	4	3	3
3	5	5	3	2	4	5	4	4
3	5	1	5	1	3	3	4	5
3	5	1	1	1	4	4	5	3
3	5	1	1	1	3	4	4	3
3	3	1	1	1	3	4	5	5
3	4	3	3	3	4	4	3	3
3	5	4	5	3	1	1	1	1
4	4	5	4	5	1	1	1	1
4	3	5	3	5	1	1	1	1
5	3	5	3	3	1	1	1	1
5	4	3	4	3	1	1	1	1
4	3	4	3	3	1	1	1	1
5	5	5	3	5	5	3	4	5
4	3	4	4	3	5	5	5	4
4	4	5	4	4	5	5	5	5
5	4	3	4	3	5	5	5	5
5	5	5	4	3	5	5	5	5
4	3	4	3	3	5	5	5	5
5	3	3	5	5	5	5	5	5
5	5	3	5	5	5	5	5	5
5	4	3	3	4	5	5	5	5
4	4	3	5	4	5	5	5	5
5	5	4	4	5	5	5	5	5
5	4	5	5	3	5	5	5	5
5	4	5	5	5	4	5	3	5
5	5	5	5	5	5	4	4	4
5	4	5	5	5	4	5	3	3
3	3	5	5	5	4	5	4	3
3	3	5	5	5	4	4	4	3
3	3	5	5	5	4	5	5	5
3	3	5	5	5	3	3	3	4
3	3	5	5	5	4	4	3	5
3	3	5	5	5	5	4	5	3
3	3	5	5	5	5	4	4	3
3	3	5	5	5	5	4	5	3
3	3	5	5	5	4	3	4	3
3	3	5	5	5	4	4	3	5
3	3	5	5	5	5	5	3	4
3	3	5	5	5	1	1	1	1
3	3	5	5	5	1	1	1	1
3	3	5	5	3	1	1	1	1
4	5	3	3	4	1	1	1	1
5	5	4	3	5	1	1	1	1
4	5	4	5	3	1	1	1	1
5	5	3	3	4	1	1	1	1
4	5	4	5	3	5	5	3	3
4	5	3	5	3	4	5	4	5
5	5	4	3	5	5	3	5	4
5	5	5	5	4	3	4	4	3
5	5	4	4	5	4	5	3	4

4	5	5	4	3	5	4	3	5
5	5	4	4	4	3	4	4	5
5	5	4	4	5	3	3	5	4
4	5	5	3	5	4	3	5	4
5	5	4	4	3	3	5	3	3
5	5	5	3	3	3	4	5	4
5	5	4	3	5	3	2	1	2
4	5	4	5	4	1	1	3	1
4	5	3	3	4	3	2	1	1
5	5	5	5	5	2	1	2	2
4	5	3	3	4	4	5	3	5
5	5	3	3	3	5	5	4	4
5	5	5	5	3	3	3	3	5
5	5	3	3	3	4	4	5	3
4	5	5	5	4	5	4	4	3
5	5	4	5	3	4	5	4	5
5	3	3	3	5	4	4	3	4
5	3	4	3	4	3	3	5	4
5	4	3	3	4	3	4	4	3
4	5	4	3	5	3	5	5	4
4	4	4	3	3	5	3	3	5
5	4	4	5	3	1	1	1	1
4	3	3	3	5	1	1	1	1
5	3	4	5	4	1	1	1	1
5	5	4	4	4	1	1	1	1
5	4	4	3	4	1	1	1	1
5	4	3	3	3	1	1	1	1
5	5	3	3	4	1	1	1	1
4	5	3	4	4	5	3	4	5
4	5	4	3	4	3	5	4	5
5	5	5	5	3	3	3	3	3
4	5	4	5	4	3	3	3	3
4	5	5	3	4	4	3	5	3
5	5	4	3	5	4	4	4	4
4	5	4	3	3	5	4	3	5
4	5	5	4	5	5	3	4	3
4	5	3	5	3	4	4	3	5
4	5	3	3	5	5	3	3	5
4	5	4	5	3	3	5	2	2
4	5	5	5	3	4	5	3	2
2	2	1	2	3	1	1	3	3
1	2	2	2	1	1	1	2	3
2	1	2	3	1	1	2	2	2
2	3	1	3	2	2	1	1	2
2	3	1	3	1	2	1	2	1
1	3	2	1	3	1	1	2	1
2	1	1	1	3	3	1	2	1
1	1	2	3	2	2	1	1	2
1	2	1	3	1	2	2	2	3
2	1	2	3	2	3	1	2	1
3	3	3	3	1	2	2	3	1
3	3	2	3	2	3	1	2	3
2	1	1	3	2	3	1	1	2
1	1	2	3	2	3	2	1	2
2	3	2	3	1	1	1	2	3
1	2	2	2	2	2	1	2	2
2	2	2	2	2	3	1	2	1
2	1	1	1	2	1	2	1	3

2	1	1	1	2	3	2	3	1
1	1	2	2	1	2	2	2	2
2	1	1	2	1	2	1	2	1
2	2	2	3	1	3	1	1	2
2	3	2	3	2	1	2	3	2
2	2	2	2	1	3	2	3	1
1	3	1	3	2	2	1	2	1
1	1	1	1	2	3	1	2	3
3	3	1	1	2	2	3	4	1
5	5	2	2	1	1	5	5	5
4	4	1	3	1	2	4	5	4
4	4	1	1	1	1	4	5	4
4	4	2	2	2	2	3	4	3
5	5	3	1	1	2	5	4	3
1	2	1	2	1	2	4	1	4
4	4	3	2	1	3	2	3	2
4	3	3	5	3	4	4	3	5
4	3	3	3	1	5	3	3	5
4	3	4	5	3	3	5	2	2
4	3	5	5	3	4	5	3	2
2	2	1	2	3	1	1	3	3
1	2	2	2	1	1	1	2	3
2	1	2	3	1	1	2	2	2
4	2	2	1	3	3	2	4	1
2	3	2	2	2	2	1	1	1
1	2	1	1	2	2	1	1	2
2	2	2	3	2	3	2	1	1
2	3	1	2	2	3	2	3	1
5	4	5	3	1	4	4	5	4
5	4	3	3	1	3	5	4	5
5	4	3	5	1	4	4	5	5
3	4	5	5	1	4	3	5	3
3	5	5	3	2	3	4	3	3
5	3	5	3	2	4	5	4	4

PP1	PP2	PP3	PP4
1	1	1	1
1	1	1	1
1	1	1	1
1	1	1	1
1	1	1	1
1	1	1	1
1	1	1	1
1	1	1	1
5	4	3	4
3	5	4	4
4	5	4	4
3	5	5	4
5	4	4	5
4	5	4	4
3	5	4	5
4	5	5	5
4	5	5	4
5	4	3	4
3	5	5	5
3	5	5	4
3	5	5	4
5	4	3	4
4	5	5	5
5	5	4	5
4	5	3	4
3	5	4	4
3	5	3	5
5	4	4	5
5	5	5	4
3	5	3	5
5	4	3	5
4	5	3	4
4	4	3	4
3	4	3	5
3	5	5	4
4	5	4	4
5	1	4	1
1	4	1	1
1	4	5	1
1	1	1	1
4	5	5	1
1	1	1	1
3	4	1	4
1	1	1	4
4	5	5	4
5	4	4	5
3	5	5	5
5	4	5	4
2	2	3	3
4	4	3	4
3	4	3	3
4	4	3	5
4	3	5	4
5	4	5	5
3	3	3	3
4	3	4	4
4	5	3	4

5	4	5	4
3	3	4	3
3	4	4	4
5	5	5	4
5	4	5	4
3	4	3	4
4	4	5	4
3	4	4	3
5	5	4	5
4	4	5	4
4	5	4	4
4	4	4	4
5	4	4	5
3	2	1	2
1	2	3	1
2	2	3	2
2	2	3	1
3	1	3	2
1	1	3	2
1	1	3	2
3	1	1	2
1	2	2	1
2	2	2	1
3	1	2	1
1	1	2	1
2	2	3	1
3	1	2	2
3	2	1	1
2	2	2	2
2	1	1	2
3	2	2	1
3	1	2	1
1	1	3	1
1	1	2	1
1	1	2	2
1	2	1	1
3	2	2	1
1	2	3	1
3	2	1	2
2	1	2	1
1	2	2	1
1	1	1	1
1	2	1	2
2	1	3	2
3	2	3	2
1	1	3	1
1	1	1	1
1	1	1	1
3	2	3	1
2	1	2	2
2	2	1	2
2	1	3	1
1	1	3	1
1	1	1	2
5	5	5	5
5	5	5	5
5	5	5	5
5	5	5	5

5	5	5	5
5	5	5	5
5	5	5	5
5	5	5	5
5	5	5	5
5	5	5	5
5	5	5	5
5	5	5	5
5	5	5	5
5	5	5	5
5	5	5	5
5	5	5	5
5	5	5	5
5	5	5	5
5	5	5	5
5	5	5	5
5	5	5	5
5	5	5	5
5	5	5	5
1	2	2	1
2	2	3	2
2	2	2	2
2	2	3	2
1	1	2	2
2	2	1	1
3	3	2	2
2	3	3	3
3	2	2	2
3	3	1	2
1	3	1	2
1	2	1	1
1	2	3	2
3	1	2	1
2	2	1	2
2	1	1	2
2	2	1	2
3	1	2	1
2	1	1	2
3	2	1	1
2	2	2	1
4	5	4	5
4	4	4	5
4	5	4	5
4	5	4	5
4	5	5	5
4	5	4	4
4	5	3	5
4	5	5	4
4	5	5	4
4	4	5	5
3	3	4	3
3	4	4	4
5	5	5	4
5	4	5	4
3	4	3	4
4	4	5	4
3	4	4	3
5	5	4	5

4	4	5	4
4	5	4	4
4	4	4	4
5	4	4	5
4	5	4	5
5	5	5	4
3	4	5	4
3	5	4	5
2	5	5	4
3	3	3	4
2	4	4	4
4	4	4	3
5	5	4	4
3	3	4	4
4	5	3	4
5	5	5	2
3	1	2	1
2	1	1	2
3	2	1	1
2	2	2	1
4	5	4	5
4	4	4	5
4	5	4	5
4	3	4	4
4	5	3	4
5	4	5	4
4	5	4	5
3	4	5	5
2	1	1	2
3	2	2	2
1	1	1	2
1	3	2	2
2	3	2	1
2	2	1	1
